# Clinical relevance of biomarker discordance between primary breast cancers and synchronous axillary lymph node metastases

**DOI:** 10.1007/s10585-023-10214-w

**Published:** 2023-07-01

**Authors:** Slavica Janeva, Toshima Z. Parris, Ellen Krabbe, Marie Sundquist, Per Karlsson, Riccardo A. Audisio, Roger Olofsson Bagge, Anikó Kovács

**Affiliations:** 1 Sahlgrenska Breast Center, Department of Surgery, Sahlgrenska University Hospital, Region Västra Götaland, Gothenburg, Sweden; 2 Department of Clinical Pathology, Institute of Biomedicine, Sahlgrenska Academy, University of Gothenburg, Gothenburg, Sweden; 3 Department of Oncology, Institute of Clinical Sciences, Sahlgrenska Academy, University of Gothenburg, Gothenburg, Sweden; 4 Sahlgrenska Center for Cancer Research, Sahlgrenska Academy, University of Gothenburg, Gothenburg, Sweden; 5 Department of Surgery, Kungälv Hospital, Region Västra Götaland, Kungälv, Sweden; 6 Department of Surgery, Kalmar County Hospital, Kalmar, Sweden; 7 Department of Oncology, Sahlgrenska University Hospital, Region Västra Götaland, Gothenburg, Sweden; 8 Department of Surgery, Institute of Clinical Sciences, Sahlgrenska Academy, University of Gothenburg, Gothenburg, Sweden; 9 Wallenberg Centre for Molecular and Translational Medicine, University of Gothenburg, Gothenburg, Sweden; 10 Department of Clinical Pathology, Sahlgrenska University Hospital, Region Västra Götaland, Gothenburg, Sweden

**Keywords:** Breast cancer, Synchronous lymph node metastasis, Discordance, Biomarkers, Surrogate subtype

## Abstract

Clinical decision-making for patients with breast cancer (BC) is still primarily based on biomarker characteristics of the primary tumor, together with the evaluation of synchronous axillary lymph node metastasis (LNM). In this study, we investigated the prevalence of discordance in the biomarkers and surrogate subtyping between the primary BC and the LNM, and whether subsequent changes would have altered clinical treatment recommendations. In this retrospective study, 94 patients treated for unifocal primary BC and synchronous LNM at Sahlgrenska UniversityHospital during 2018 were included. Estrogen (ER) and progesterone (PR) receptor, Ki67, and HER2 status were assessed in the primary tumor and LNM using immunohistochemistry. Discordances between the primary tumor and the LNM were analyzed for each individual biomarker and surrogate subtyping. The concordance between the primary tumor and the LNM for ER, PR, Ki67, and HER2 status was 98.9%, 89.4%, 72.3%, and 95.8%, respectively. Discordance in surrogate subtyping was found in 28.7% of the tumors and matched LNMs, the majority (81.5%) of which changed to a more favorable subtype in the LNM; most commonly from Luminal B to Luminal A (48.6%). No changes in surrogate subtyping were detected where ER or HER2 status changed from negativity in the BC to positivity in the LNM, thereby showing no additional value in performing immunohistochemistry on the LNM from a treatment decision-making perspective. However, large studies need to be performed that test both the primary BCs and synchronous LNMs for more accurate diagnostics.

**Supplementary Information:**

The online version contains supplementary material available at 10.1007/s10585-023-10214-w.

## Introduction

Treatment-decisions for patients with breast cancer (BC) are based on biomarker characteristics of the primary BC. Although gene expression tests, e.g. Oncotype DX, Mammaprint and Prosigna, are frequently used for BC, classification of surrogate subtyping based on tumor grade and immunohistochemical analysis of the estrogen (ER) and progesterone (PR) receptors, proliferation factor Ki67, and human epidermal growth factor receptor − 2 (HER2) still remains the routine method for tumor classification. Synchronous axillary lymph node metastasis (LNM) is the most important predictor of recurrence and overall survival [1–3]. In the event of LNM, treatment planning might be altered (e.g. surgery, hormonal therapy, chemotherapy, anti-HER2 treatment and radiotherapy). Studies have confirmed discordance between the primary BC and the LNM for individual biomarkers, as well as the surrogate subtypes. Discordance rates of 3–30% and 3–32% have been reported for ER and PR, respectively [4–9], while 0–14% discordance has been reported for HER2 [8, 10, 11]. Moreover, conflicting results have been found for Ki67, with both higher [4, 5, 12, 13] and lower [14] Ki67 values in the LNM compared to the primary site. When the biomarker profile of the LNM was used to determine the surrogate subtype, 11–46% of the primary tumors and the lymph nodes were discordant [10, 14, 15]. However, few studies have investigated the clinical implications of discordant surrogate subtyping between the BC and the lymph node metastasis.

In this study, we evaluated [1] the prevalence of discordance in BC biomarker status and surrogate subtyping between the primary tumor and the LNM, and [2] whether subsequent changes would have altered clinical treatment recommendations.

## Materials and methods

### Study population

In this retrospective study, patients with BC at Sahlgrenska University Hospital (Gothenburg, Sweden) during 2018 were screened for inclusion. Inclusion criteria were unifocal primary BC and synchronous LNM. Patients could have a clinically positive axillary lymph node, preoperatively verified by cytology or biopsy, or the metastasis could be a positive sentinel lymph node (SLN) identified postoperatively. Exclusion criteria were multifocal BC, occult BC, bilateral BC, recurrent disease, patients that received neoadjuvant treatment, and distant metastasis known at the time of the postoperative multidisciplinary treatment conference. Patient data, medical records and pathology reports were retrieved from the Swedish National Breast Cancer Register and local hospital medical records. All treatment recommendations were determined according to applicable Swedish guidelines for 2018. The Regional Ethical Review Board in Gothenburg, Sweden (479 − 18) approved this study.

### Immunohistochemistry

The biomarker status (ER, PR, Ki67 and HER2) was retrieved from the original pathology reports for the 94 primary breast carcinomas operated in 2018, which were analyzed in the routine pathology workflow by two experienced, board-certified pathologists, subspecialized in breast pathology. Additional immunohistochemistry was performed for ER, PR, HER2, and Ki67 with 4 μm full-face sections from formalin-fixed, paraffin-embedded (FFPE) tissue of the axillary LNMs using the same staining protocol and antibodies as for the primary breast carcinomas (Supplementary Table 1). According to the Swedish national guidelines applicable for 2018, ER and PR were considered positive when there was ≥ 10% immunostaining in neoplastic cells and Ki67 was considered high with ≥ 20% immunostaining. Samples with HercepTest scores of 2 + and 3 + were confirmed for HER2 amplification using silver in situ hybridization (SISH). Receptor-based surrogate subtypes (Luminal A, Luminal B HER2-, Luminal B HER2+, Non-luminal HER2+, and triple-negative breast cancer [TNBC]) for the primary BC and synchronous LNM were determined according to grade (in the primary BC, not the LNM) and biomarkers using national guidelines, which were equivalent to the 2017 St. Gallen molecular classification for Ki67 and HER2, but not for ER and PR (St. Gallen: ER/PR immunopositivity ≥ 1% in neoplastic cells) [16].

### Statistical analysis

All statistical analyses were performed using R/Bioconductor (v4.1.1) with two-sided p-values with a cut-off of 0.05. Descriptive statistics for continuous variables were presented with median and quartiles, as well as percentages and frequencies for categorical variables. Concordance between the BC and the LNM for categorical characteristics was explored by two-way crosstabs. The tableone R script (v0.13.2) was used to evaluate the relationship between clinicopathologic features and BC/LNM with Chi-square test for categorical variables [17, 18]. Cohen kappa statistic with 95% two-sided confidence intervals (CI) was calculated to estimate the overall agreement between the biomarker status for ER, PR, HER2, and Ki67 in the BC and the LNM using the irr package (v0.84.1) in R [19]. Kappa-values > 0.8, between 0.6 and 0.8, between 0.4 and 0.6, < 0.4, and < 0.2 were classified as very good, good, moderate, fair, and poor agreement, respectively. The correlation between ER, PR, HER2, and Ki67 status in the BC and the LNM was determined using linear regression with the ggplot2 package (v3.4.0) in R [20]. Sankey diagrams were constructed to illustrate potential changes in biomarker status and surrogate subtyping between the BC and the LNM using the alluvial package (v0.2-0) in R [21]. The ggsurvfit package (v0.1.0) in R was used for construction of Kaplan-Meier plots for disease-free survival (DFS) and overall survival (OS) [22]. DFS was defined as the time from surgery to either local recurrence/distant metastasis or death, while OS was defined as the time from surgery to death of any cause. End of follow-up was October 14, 2022.

## Results

### Patient characteristics

**Fig. 1:**
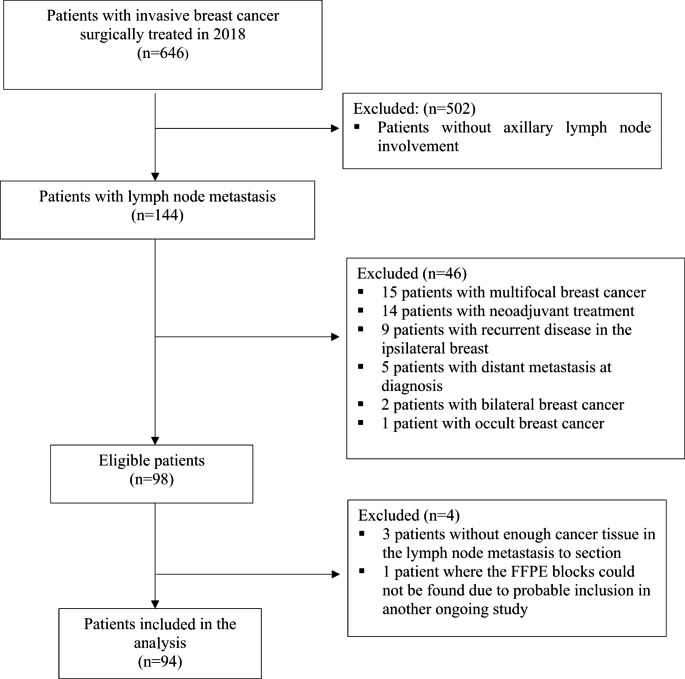
Flowchart of patients with invasive breast cancer and synchronous lymph node metastasis surgically treated in 2018

In 2018, 144 patients with unifocal BC and synchronous axillary LNM were treated for invasive BC at Sahlgrenska University Hospital (Gothenburg, Sweden). After removing patients fulfilling the exclusion criteria, 94 patients (93 women, 1 man) were included in the study (Fig. 1). The median age was 65.5 years (interquartile range [IQR] 53.5–76.0 years). Mastectomy (51.1%) was the most common surgery performed in the breast and an upfront sentinel lymph node biopsy (SLNB) followed by an axillary lymph node dissection (ALND) at a later point was the most common surgery in the axilla (41.5%). Moreover, the majority of patients received adjuvant endocrine therapy (92.6%), radiotherapy (87.2%), and chemotherapy (60.6%; Table 1).

**Table 1:** Characteristics of patients primarily surgically treated for primary breast cancer and synchronous lymph node metastasis at Sahlgrenska University Hospital (Gothenburg, Sweden) in 2018

No. of patients (n)	94
**Age, years (median [IQR])**	65.5 [53.5, 76.0]
**Disease-free survival time, months (median [IQR])**	50.0 [46.3, 55.0]
**Overall survival time, months (median [IQR])**	50.5 [48.0, 55.0]
**No of events, recurrence (local, distant; %)**	16 (17.0)
**No of events, death (%)**	12 (12.8)
**No. of patients with both events**	5 (5.3)
**Surgery, breast (%)**	
Mastectomy	48 (51.1)
Breast conserving surgery	46 (48.9)
**Surgery, axilla (%)**	
Sentinel lymph node biopsy (SLNB) only Axillary lymph node dissection (ALND)	25 (26.7) 30 (31.9)
SLNB + ALND^1^	39 (41.5)
**Breast cancer features**	
**Size, mm (median [IQR])**	31.5 [20.0, 47.8]
**Pathologic tumor size (%)**	
pT1	23 (24.5)
pT2	50 (53.2)
pT3	21 (22.3)
**Histological type (%)**	
Ductal	73 (77.7)
Lobular	8 (8.5)
Tubular	5 (5.3)
Tubulolobular	1 (1.1)
Mixed	7 (7.5)
**Histological grade, NHG (%)**	
NHG 1	8 (8.5)
NHG 2	53 (56.4)
NHG 3	33 (35.1)
**Lymphovascular invasion (%)**	
Yes	67 (71.3)
No	27 (28.7)
**Lymph node features**	
**Size, mm (median [IQR])**	9.0 [4.0, 13.0]
**Nodal status (%)**	
pN_micro_	7 (7.4)
pN1	62 (66.0)
pN2	20 (21.3)
pN3	5 (5.3)
**Adjuvant therapy received**	
Endocrine therapy (%)	87 (92.6)
Chemotherapy (%)	57 (60.6)
Trastuzumab (%)	8 (8.5)
Radiotherapy (%)	82 (87.2)

^1^Sentinel lymph node biopsy was performed during primary surgery with additional axillary lymph node dissection at a later occasion

ALND = axillary lymph node dissection, IQR = interquartile range, NHG = Nottingham histologic grade, SLNB = sentinel lymph node biopsy

### Concordance in biomarker status and subtyping between the breast cancers and LNMs

The majority of the BCs and LNMs were ER- and PR-positive (BCs: 91.5% for ER and 78.7% for PR; LNMs: 90.4% for ER and 74.5% for PR) and HER2-negative (BCs: 89.4%; LNMs: 93.6%). For Ki67, 55.3% of the BCs, but only 36.2% of the LNMs had high expression (≥ 20%). Luminal A was the most common subtype for both BCs (43.6%) and LNM (61.7%; Table 2).

**Table 2:** Biomarker characteristics and subtypes in the primary breast cancer and synchronous lymph node metastasis in patients surgically treated at Sahlgrenska University Hospital (Gothenburg, Sweden) in 2018

	Breast cancer (n = 94)	Lymph node (n = 94)	p-value
**ER (%)**			1.0
positive^1^	86 (91.5)	85 (90.4)	
negative^2^	8 (8.5)	9 (9.6)	
**PR (%)**			0.61
positive^1^	74 (78.7)	70 (74.5)	
negative^2^	20 (21.3)	24 (25.5)	
**Ki67 (%)**			0.01
high^3^	52 (55.3)	34 (36.2)	
low^4^	42 (44.7)	60 (63.8)	
**HER2 (%)**			0.43
positive^5^	10 (10.6)	6 (6.4)	
negative^6^	84 (89.4)	88 (93.6)	
**Subtype (%)**			0.06
Luminal A	41 (43.6)	58 (61.7)	
Luminal B HER2-	37 (39.4)	24 (25.5)	
Luminal B HER2+	9 (9.6)	4 (4.3)	
Non-luminal HER2+	1 (1.1)	2 (2.1)	
TNBC	6 (6.4)	6 (6.4)	

^1^ if ≥ 10% immunostaining in neoplastic cells

^2^ if < 10% immunostaining in neoplastic cells

^3^ with ≥ 20% immunostaining in neoplastic cells

^4^ with < 20% immunostaining in neoplastic cells

^5^ amplified with silver in situ hybridization

^6^ HercepTest scoring 0 or 1 + or not amplified with silver in situ hybridization

ER = estrogen receptor, PR = progesterone receptor, HER2 = human epidermal growth factor 2, TNBC = triple-negative breast cancer

**Fig. 2:**
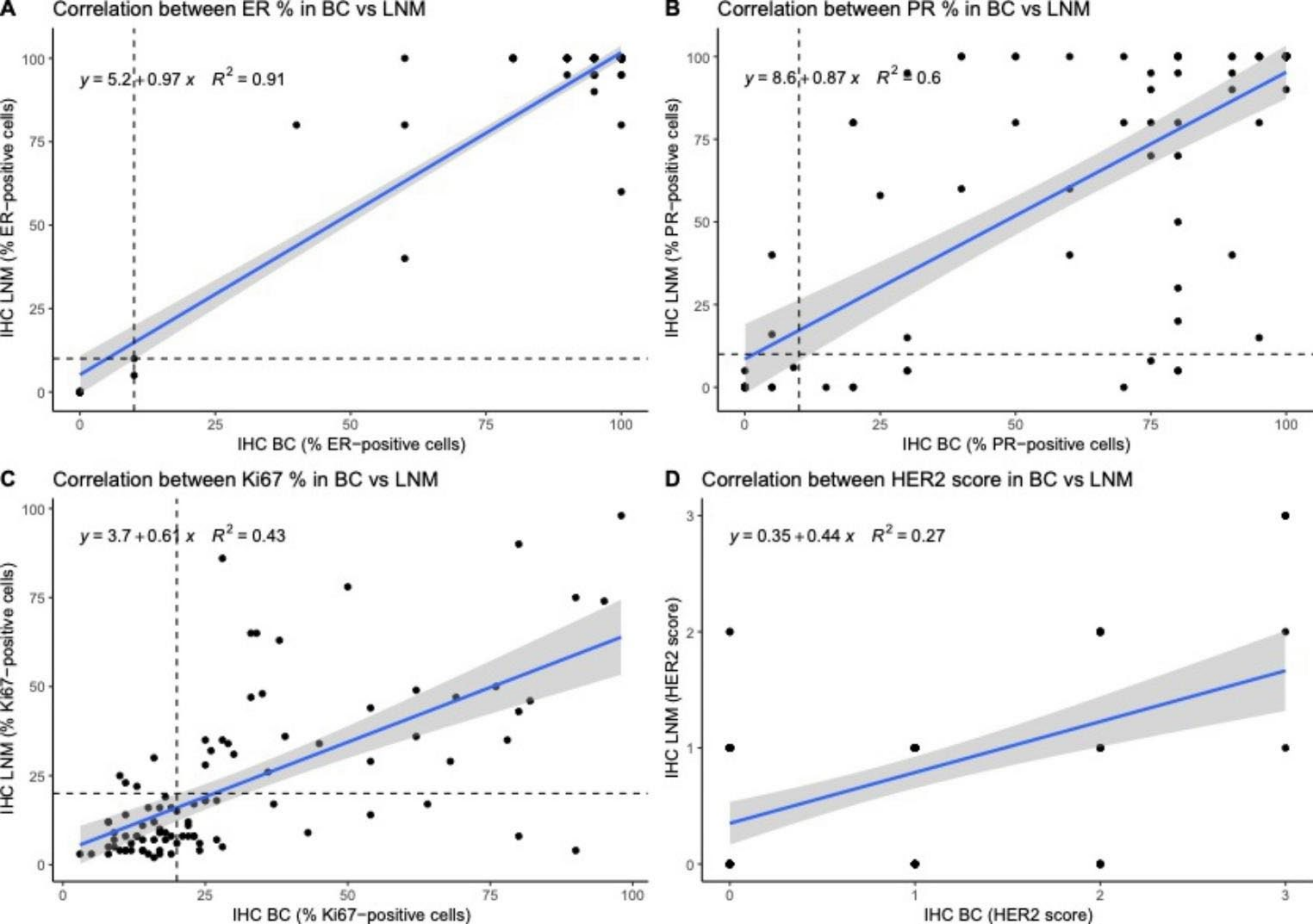
Correlation between biomarkers in the breast cancer and the paired synchronous lymph node metastasis. For ER, PR, and Ki67, correlation was calculated with consideration to the percentage of expression in neoplastic cells. For HER2, correlation was calculated for HER2-scoring (0, 1+, 2 + and 3+) BC = primary breast cancer, LNM = synchronous lymph node metastases

**Fig. 3:**
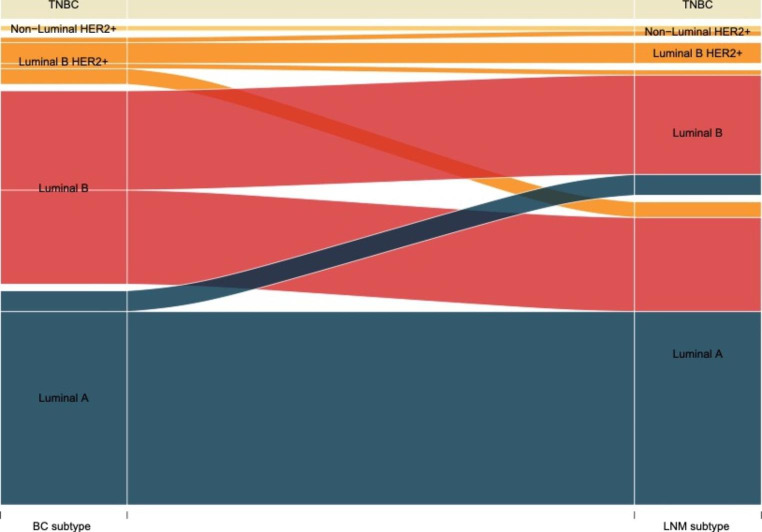
Sankey diagram for subtype changes between the primary breast cancer (BC) and synchronous lymph node metastasis (LNM).

The concordance in biomarker expression was then evaluated between the primary BC and the LNM, thereby showing concordance rates of 98.9% for ER (ER-positive: 90.4%; ER-negative: 8.5%), 95.8% for HER2 (HER2-positive: 6.4%; HER2-negative: 89.4%), 89.4% for PR (PR-positive: 71.3%; PR-negative: 18.1%), and 72.3% for Ki67 (Ki-67 high: 31.9%; Ki-67 low: 40.4%; Table 3). Ki67 changed from high in the BC to low in the LNM for 22 patients (23.4%), while changes from low to high status were only observed for four BC/LNM pairs (4.3%). Correlation analysis revealed that the strongest positive correlation between immunohistochemical staining in the primary tumor and the LNM was for ER status (R^2^ = 0.91), while HercepTest-scoring showed the weakest correlation (R^2^ = 0.27; Fig. 2). In total, 67 (71.3%) of BC/LNM pairs were concordant for surrogate subtyping, i.e., 37 patients in the Luminal A group, 19 patients in the Luminal B/HER2-negative group, 4 patients in the Luminal B/HER2-positive group, 1 patient in the Non-luminal/HER2-positive group, and 6 patients in the TNBC group (Table 4). Intriguingly, 22 of the 27 discordant LNMs (81.5%) were classified as a more favorable subtype, e.g., 48.6% of the Luminal B HER2- BCs had a LNM of Luminal A subtype. The varying widths of the flows in the Sankey diagram provide insights into the relative distribution of different subtypes of breast cancer and synchronous lymph node metastasis. The diagram visually highlights the lack of specific transitions between ER-negative primary tumors with ER-positive lymph node metastases. Furthermore, it clearly demonstrates that there are no HER2-negative primary tumors with simultaneous HER2-positive lymph node metastases (Fig. 3). Concordance of HercepTest scoring and distribution of the SISH-amplified tumors are presented in Supplementary Table 2. Both the 10 HER2-amplified BCs and six amplified LNMs were equally distributed in the HercepTest 2 + and 3 + scoring groups. An evaluation of Ki67 status in patients with micro metastasis (< 2 mm) in the LNM revealed that all paired samples (n = 7) were low in Ki67/Luminal A in both the BC and the paired LNM (Supplementary Table 3).

**Table 3:** Concordance between immunohistochemical expression of ER, PR, Ki67, and HER2 in the primary breast cancer and synchronous lymph node metastasis. ER = estrogen receptor, PR = progesterone receptor, HER2 = human epidermal growth factor 2

	Breast cancer	Lymph node	n = 94	%	Cohen´s Kappa^2^	Strength of agreement^2^		p-value	
ER^1^	pos	pos	85	90.4	0.935	very good	<<0.001		
	neg	neg	8	8.5					
	pos	neg	1	1.2					
	neg	pos	0	0.0					
PR^1^	pos	pos	67	71.3	0.704	good	≤ 0.001		
	neg	neg	17	18.1					
	pos	neg	7	7.4					
	neg	pos	3	3.2					
Ki67^1^	high	high	30	31.9	0.463	moderate	≤ 0.001		
	low	low	38	40.4					
	high	low	22	23.4					
	low	high	4	4.3					
HER2^1^	pos	pos	6	6.4	0.728	good	≤ 0.001		
	neg	neg	84	89.4					
	pos	neg	4	4.3					
	neg	pos	0	0.0					

^1^ER and PR are considered to be positive with ≥ 10% and Ki67 is considered high with ≥ 20% immunostaining, respectively. HER2 is considered to be positive with HercepTest scored 3 + or confirmed HER2 amplification using SISH (silver in situ hybridization) testing

^2^Cohen’s kappa κ-values: >0.8 indicate very good agreement, between 0.6 and 0.8 indicate good agreement, between 0.4 and 0.6 indicate moderate agreement, < 0.4 indicate fair agreement, < 0.2 indicate poor agreement

**Table 4:** Concordance in surrogate subtypes between the primary breast cancer and synchronous lymph node metastasis

		Lymph node metastasis					
		**Luminal A**	**Luminal B HER2-**	**Luminal B HER2+**	**Non-luminal HER2+**	**TNBC**	Total
**Breast cancer**	**Luminal A**	37	4	0	0	0	41
	**Luminal B HER2-**	18	19	0	0	0	37
	**Luminal B HER2+**	3	1	4	1	0	9
	**Non-luminal HER2+**	0	0	0	1	0	1
	**TNBC**	0	0	0	0	6	6
	Total	58	24	4	2	6	94

### Survival analysis based on concordance in biomarker status and subtyping

Survival analysis showed no significant differences in neither overall survival (OS) nor disease-free survival (DFS) between the concordant and discordant groups for biomarker status and subtyping (Supplementary Fig. 1). Median DFS was 50.0 months (IQR 46–55 months), while median OS was 50.5 months (IQR 48–55 months)”. Of the 16 patients with local/distant recurrence, 10 were biopsy verified and assessed with immunohistochemistry (Supplementary Table 4). Four of the ten patients were concordant for subtype in all three locations, i.e., the primary BC, the synchronous LNM, and the recurrent metastasis.

## Discussion

In this retrospective study we observed varying discordance in biomarker expression between the primary BC and synchronous LNM for ER (1.1%), PR (10.6%), Ki67 (27.7%), and HER2 (4.2%). Furthermore, surrogate subtyping was discordant in 28.7% of the BC-LNM pairs. The majority (81.5%) of discordant subtyping changed to a more favorable subtype in the LNM, frequently from Luminal B HER2- to Luminal A (48.6%). Survival analysis demonstrated no difference in DFS or OS between concordant and discordant patients, for neither the individual BC biomarkers nor surrogate subtypes. To the best of our knowledge, these comparisons have not been previously described. However, for any conclusions to be drawn, much larger datasets adjusting for patient and tumor characteristics are needed. The data presented here should be seen as having descriptive purposes only.

Compared to similar studies, we found that discrepancies in ER, PR, and HER2 status were in the lower range in the present study. However, discordant Ki67 status has not been sufficiently studied in BC compared to the other three biomarkers. For ER status, Jensen et al. [23] showed a discordance of 1.6%, while Aitken et al. [9] and Kinoe et al. [5] reported discordance rates as high as 28%. In line with the present study, Zhao and colleagues [7] demonstrated relatively low discordance rates for PR (11.1%). On the other hand, two studies showed comparably different discordance rates for HER2 (3.9–43.3%) and Ki67 (4.4–43.3%), with our results falling in the lower and middle ranges for each biomarker, respectively [5, 24].

Relatively few studies investigated changes in subtype in the primary BC and matched LNM. Here, we show that 28.7% of matched BCs and LNMs were discordant for surrogate subtype. Subtyping was predominantly more favorable in the LNM than the BC sample, with 48.5% of the discrepancies constituting changes from Luminal B HER2- (BC) to Luminal A (LNM). This is in agreement with other studies, where Bonin et al. [14] showed a subtype discordance of 46% and Luminal B to a Luminal A change in 21 of 45 Luminal B BCs (53%). Mandó et al. [15] showed a subtype change in 28% of the tumors with the most frequent alteration from Luminal B to Luminal A (36.4%). These findings are in contrast to several reports showing changes to a more aggressive subtype in the LNM [9, 10, 25, 26], which is also in line with studies that only evaluated Ki67 (i.e. low Ki67 levels in the BC and high Ki67 levels in the LNM) [26–28]. In this study, we only observed 4.3% of the cases where Ki67 status changed from low to high in the LNM, whereas 23.4% of cases changed from high to low in the LNM. However, it is difficult to compare different studies due to varying cut-off values for Ki67. It has also been highlighted that the size of the LNM has an impact on Ki67 detection. Since it is difficult to identify Ki67 hotspots in small metastases (< 2 mm), the Ki67 index in the LNM and the primary BC may not correlate. In our study, the median size of the LNM was relatively large (at median 9.0 mm). We also conducted a subgroup analysis of the smallest LNMs (micro metastases < 2 mm), thereby demonstrating no changes in Ki67 status between the BC and the LNM, with a low Ki67 in all pairs (Luminal A/Luminal A).

Aitken et al. [9] postulated that ER-positive patients that fail to respond to endocrine therapy may possibly be linked to tumor progression from ER-positive BC to ER-negative LNM. Perhaps such patients would benefit from additional chemotherapy instead. Here, one discordant ER-positive patient lost ER expression in the LNM. This patient had a Luminal B HER2 + surrogate subtype, with ER expression of 10% in the breast (ER-positive according to the national Swedish guidelines for 2018) and non-luminal HER2 + surrogate subtype with ER expression of 5% in the LNM (ER-negative according to the Swedish guidelines). This patient received endocrine therapy as well as combination treatment with trastuzumab and chemotherapy. In addition, no events were reported for this patient.

Recently, new perspectives regarding HER2-targeted treatment in so called “HER2-low” BCs (i.e., patients with HercepTest scores 1 + and 2 + without amplification) have occurred. There are studies showing no clinical benefit with traditional HER2-targeted agents in HER2-low BCs [29, 30], but promising results have been presented regarding HER2-directed antibody drug conjugates with chemotherapeutics in the metastatic setting [31, 32]. Tarantino and colleagues [33] recently demonstrated a significant discordance in terms of HER2-low expression between primary tumors and matched advanced-stage biopsies, with enrichment in HER2-low tumors in the advanced setting. This enrichment was mainly related to a shift of HER2-0 primary tumors into HER2-low tumors, in the advanced setting, with the opposite trend (shift from HER2-low towards HER2-0) being less frequent. In our study, we presented 17 patients where the HercepTest score changed from 0 to HER2-low status in the LNM. Although synchronous axillary lymph node metastasis is not considered as distant metastasis, one hypothesis could be that these patients might benefit from treatment with HER2-directed antibody drug conjugates. Further studies need to be performed to investigate this in the clinical setting.

BCs are highly heterogeneous, and several studies have postulated that the most aggressive tumor cell clones metastasize to the lymph nodes. However, we did not observe a substantial worsening in subtype from the primary cancer to either the LNM or the biopsy confirmed as metastatic disease. Despite recorded discrepancies in biomarker status between the primary BC and LNM, no changes from ER-negativity in the BC to ER-positivity in the LNM were found. To our knowledge only Dikicioglu et al. [13] showed similar results, although their sample size was relatively small (*n =* 22*)*. In a study by Jensen et al. [23], all samples were concordant for HER2 status. Kuncman et al. [34] had similar findings, but they did not include any cases with HER2 score 2+. To our knowledge, this is the first study to report the lack of changes from ER-negative or HER2-negative BCs to ER positive or HER2-positive LNMs, which would theoretically have led to the recommendation of additional treatment. Despite affecting few patients, other studies have shown discordances in both ER and HER2 status that theoretically would have led to additional endocrine therapy or HER2-targeted treatment [5, 8–10, 14, 23, 25, 34, 35].

In the present study, we could not show any significant clinical value of using biomarker status or surrogate subtyping in the synchronous LNMs as a means of recommending additional endocrine therapy or targeted HER2 therapy for patients with BC. However, the study findings must be viewed with caution due to the retrospective study design and the limited number of patients. Although many of the studies investigating this topic have cohorts of around 100 patients, the sample size is still considered to be relatively low. It was especially difficult to compare studies due to differences in guidelines from different years and cut-off values for the BC biomarkers and surrogate subtypes. In addition, many of the studies assessed biomarker expression using tissue microarrays (TMA), which might not fully reflect tumor heterogeneity in full, while full-face FFPE sections were used in the present study. Another limitation of the present study is the lack of molecular data (e.g. Oncotype DX, PAM50/Prosigna) for the BCs and LNMs. Although genetic assays are more frequently used in daily clinical practice, IHC remains the standard method for BC classification. Additional treatment for BC based on biomarker status in the LNM, or treatment based on the LNM instead of the primary BC, is a topic for further studies where determination of molecular subtypes preferably should be performed using multi-gene assays.

## Conclusion

In this study, no changes in ER or HER2 status from negativity in the primary BC to positivity in the LNM were detected. Therefore, immunohistochemical assessment of the LNM would not have led to additional treatments using endocrine or HER2-targeted therapies. To explore this further and enable more accurate diagnostics, multi-gene testing of larger cohorts comprised of BCs and matched LNMs need to be performed.

## Electronic supplementary material

Below is the link to the electronic supplementary material.

Supplementary Material 1

Supplementary Material 2

Supplementary Material 3

Supplementary Material 4

Supplementary Material 5

Supplementary Material 6

## Data Availability

The data supporting the findings are available upon reasonable request to the corresponding author.
