# Supplementary material for: Clinical relevance of biomarker discordance between primary breast cancers and synchronous axillary lymph node metastases
Source: Clin Exp Metastasis. 2023 Jul 1;40(4):299–308. doi: 10.1007/s10585-023-10214-w (PMC10338601; doi:10.1007/s10585-023-10214-w)
Supplement: Supplementary file 1 — Supplementary Material 1 [file 10585_2023_10214_MOESM1_ESM.docx]

**Supplementary Table 1.** Antibodies used for immunohistochemistry tissue staining of synchronous lymph node metastasis.

| **Antibody** | **Manufacturer** | **Clone** | **Dilution** | **Antigen retrieval** |
| --- | --- | --- | --- | --- |
| Estrogen receptor (rabbit) | DAKO IR084 | EP1 | Ready to use | TRS high, pH9.0 |
| Progesterone receptor (mouse) | DAKO IR068 | 636 | Ready to use | TRS high, pH9.0 |
| Ki67 (mouse) | DAKO IR626 | MIB-1 | Ready to use | TRS low, pH6.0 |
| HercepTest^1^ (rabbit) | DAKO SK001 | poly | Ready to use | TRS low, pH6.0 |

^1^Samples with HercepTest scores of 2+ or 3+ were additionally assessed with Ventana dual SISH test (silver *in situ* hybridization) for possible HER2 amplification.
