# Supplementary material for: Clinical relevance of biomarker discordance between primary breast cancers and synchronous axillary lymph node metastases
Source: Clin Exp Metastasis. 2023 Jul 1;40(4):299–308. doi: 10.1007/s10585-023-10214-w (PMC10338601; doi:10.1007/s10585-023-10214-w)
Supplement: Supplementary file 2 — Supplementary Material 2 [file 10585_2023_10214_MOESM2_ESM.docx]

**Supplementary Table 2.** Concordance of HercepTest-scoring in the breast cancer and matching axillary lymph node metastasis and number of SISH-amplified tumors.

|  |  |  | LNM |  |  |  |
| --- | --- | --- | --- | --- | --- | --- |
|  |  |  |  |  |  |  |
|  | HercepTest-scoring | 0+ | 1+ | 2+ | 3+ | Total |
|  | 0+ | 25 | 15 | 2 | 0 | 42 |
| Breast cancer | 1+ | 11 | 18 | 0 | 0 | 29 |
|  | 2+ | 5^1^ | 7^2^ | 6^3^ | 0 | 18 |
|  | 3+ | 0 | 1^4^ | 1^5^ | 3^6^ | 5 |
|  | Total | 41 | 41 | 9 | 3 | 94 |

LNM = lymph node metastasis

^1^2 amplified breast cancers

^2^1 amplified breast cancer

^3^2 amplified breast cancers, 2 matching amplified LNMs

^4^1 amplified breast cancer

^5^1 amplified breast cancer and 1 matching amplified LNM

^6^3 amplified breast cancers and 3 matching amplified LNMs
