# Supplementary material for: Clinical relevance of biomarker discordance between primary breast cancers and synchronous axillary lymph node metastases
Source: Clin Exp Metastasis. 2023 Jul 1;40(4):299–308. doi: 10.1007/s10585-023-10214-w (PMC10338601; doi:10.1007/s10585-023-10214-w)
Supplement: Supplementary file 3 — Supplementary Material 3 [file 10585_2023_10214_MOESM3_ESM.docx]

**Supplementary Table 3.** Ki67 index was compared between the primary BC and the LNM diagnosed as micrometastases* in seven patients.

| **Patients** | **Ki67**  **BC** | **Ki67**  **LNM** | **Subtype^1^** |
| --- | --- | --- | --- |
| No.1 | 16% | 2% | Luminal A / Luminal A |
| No.2 | 17% | 9% | Luminal A / Luminal A |
| No.3 | 11% | 4% | Luminal A / Luminal A |
| No.4 | 13% | 8% | Luminal A / Luminal A |
| No.5 | 12% | 4% | Luminal A / Luminal A |
| No.6 | 8% | 12% | Luminal A / Luminal A |
| No.7 | 19% | 4% | Luminal A / Luminal A |

^1^Subtype breast cancer (BC) / subtype lymph node metastasis (LNM)

*N_micro_: <2 mm
