# Supplementary figures and images for: Clinical relevance of biomarker discordance between primary breast cancers and synchronous axillary lymph node metastases
Source: Clin Exp Metastasis. 2023 Jul 1;40(4):299–308. doi: 10.1007/s10585-023-10214-w (PMC10338601; doi:10.1007/s10585-023-10214-w)

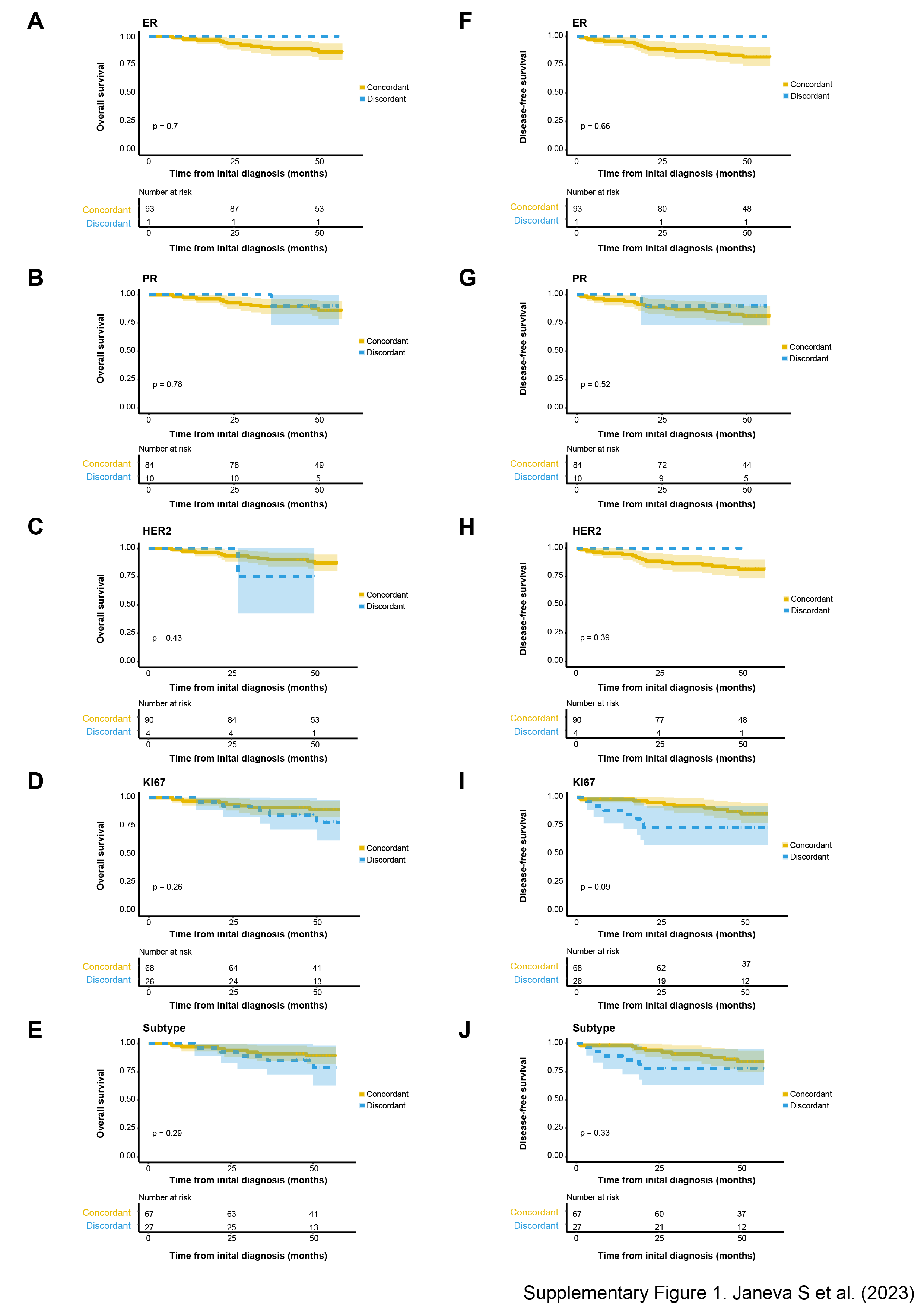

Supplement: Supplementary file 4 — Supplementary Material 4 [file 10585_2023_10214_MOESM4_ESM.tif]
