# Supplementary material for: Clinical relevance of biomarker discordance between primary breast cancers and synchronous axillary lymph node metastases
Source: Clin Exp Metastasis. 2023 Jul 1;40(4):299–308. doi: 10.1007/s10585-023-10214-w (PMC10338601; doi:10.1007/s10585-023-10214-w)
Supplement: Supplementary file 5 — Supplementary Material 5 [file 10585_2023_10214_MOESM5_ESM.docx]

**Supplementary Figure 1.** Median overall survival (OS) and median disease-free survival (DFS) for ER, PR, Ki67, HER2 and subtype in primary breast cancers and matching synchronous lymph node metastasis.
