# Supplementary material for: Clinical relevance of biomarker discordance between primary breast cancers and synchronous axillary lymph node metastases
Source: Clin Exp Metastasis. 2023 Jul 1;40(4):299–308. doi: 10.1007/s10585-023-10214-w (PMC10338601; doi:10.1007/s10585-023-10214-w)
Supplement: Supplementary file 6 — Supplementary Material 6 [file 10585_2023_10214_MOESM6_ESM.docx]

**Supplementary Table 4.** Subtype in matching breast cancer, synchronous axillary lymph node metastasis, and local recurrence/distant metastasis

|  | Breast cancer | Lymph node metastasis | Local recurrence/ |
| --- | --- | --- | --- |
|  |  |  | distant metastasis |
| Patient 1 | Luminal B HER2- | Luminal B HER2- | Luminal B HER2- |
| Patient 2 | Luminal B HER2- | Luminal B HER2- | TNBC |
| Patient 3 | Luminal B HER2- | Luminal A | Luminal A |
| Patient 4 | TNBC | TNBC | TNBC |
| Patient 5 | Luminal B HER2- | Luminal B HER2- | Luminal A |
| Patient 6 | Luminal B HER2+ | Luminal B HER2+ | Non-luminal HER2- |
| Patient 7 | Luminal B HER2- | Luminal A | Luminal B HER2- |
| Patient 8 | Luminal B HER2- | Luminal B HER2- | Luminal B HER2- |
| Patient 9 | Luminal B HER2- | Luminal B HER2- | Luminal B HER2- |
| Patient 10 | Luminal A | Luminal B HER2- | Luminal A |

TNBC = triple-negative breast cancer
